# Selective Development of Myogenic Mesenchymal Cells from Human Embryonic and Induced Pluripotent Stem Cells

**DOI:** 10.1371/journal.pone.0051638

**Published:** 2012-12-07

**Authors:** Tomonari Awaya, Takeo Kato, Yuta Mizuno, Hsi Chang, Akira Niwa, Katsutsugu Umeda, Tatsutoshi Nakahata, Toshio Heike

**Affiliations:** 1 Department of Pediatrics, Kyoto University Graduate School of Medicine, Kyoto, Japan; 2 Center for iPS Cell Research and Application (CiRA), Kyoto University, Kyoto, Japan; University of Minnesota Medical School, United States of America

## Abstract

Human embryonic stem (ES) cells and induced pluripotent stem (iPS) cells are promising sources for the cell therapy of muscle diseases and can serve as powerful experimental tools for skeletal muscle research, provided an effective method to induce skeletal muscle cells is established. However, the current methods for myogenic differentiation from human ES cells are still inefficient for clinical use, while myogenic differentiation from human iPS cells remains to be accomplished. Here, we aimed to establish a practical differentiation method to induce skeletal myogenesis from both human ES and iPS cells. To accomplish this goal, we developed a novel stepwise culture method for the selective expansion of mesenchymal cells from cell aggregations called embryoid bodies. These mesenchymal cells, which were obtained by dissociation and re-cultivation of embryoid bodies, uniformly expressed CD56 and the mesenchymal markers CD73, CD105, CD166, and CD29, and finally differentiated into mature myotubes *in vitro*. Furthermore, these myogenic mesenchymal cells exhibited stable long-term engraftment in injured muscles of immunodeficient mice *in vivo* and were reactivated upon subsequent muscle damage, increasing in number to reconstruct damaged muscles. Our simple differentiation system facilitates further utilization of ES and iPS cells in both developmental and pathological muscle research and in serving as a practical donor source for cell therapy of muscle diseases.

## Introduction

Duchenne muscular dystrophy (DMD) is the most common and well-investigated form of muscular dystrophy inherited in an X-linked recessive manner. The molecular deficits underlying this disorder are primarily involved in muscular structural integrity and result in continuous damage to the muscles due to contraction-induced mechanical stress. This damage leads to the rapid wasting of skeletal muscles and to the early deaths of affected patients [1]. Satellite cells, which are muscle-specific stem cells that reside in the muscle [2], maintain the ability of skeletal muscles to undergo self-repair and can be mobilized for reconstruction when the muscles are damaged from exercise and daily activities [3]–[5]. Although muscular regeneration occurs at a higher frequency in DMD patients than in non-affected individuals [6], it is insufficient to maintain muscle function throughout life. If satellite cells were able to restore damaged muscles more efficiently, the courses of the diseases might be less severe, as observed in mdx mice, a DMD model that is fertile and has a near-normal lifespan [1], [7], [8]. Despite extensive efforts to develop pharmacological agents to halt the clinical course of DMD, the disease still results in high mortality in patients during late adolescence.

Cell replacement therapy using extrinsic myogenic cells is one of the most promising treatment modalities for muscular dystrophies. Somatic stem cells with skeletal myogenic potential, such as myoblasts, mesenchymal stem cells, side population cells of muscles and bone marrow, pericytes, and hemangioblasts, are known to reside in various types of adult tissues. These cells regenerate diseased muscles in mdx mice; however, clinical trials involving allogeneic myoblast transplantation in DMD patients have not obtained satisfactory results because of immune rejection, rapid death, and the limited migration of transplanted myoblasts [9]–[11].

Embryonic stem (ES) cells are totipotent stem cells derived from blastocysts [12], [13] and possess considerable advantages over somatic stem cells. Because ES cells have theoretically unlimited proliferative capacity, they could be a reliable cell source for regeneration therapy, provided that an effective myogenic differentiation protocol is established. Moreover, recently established induced pluripotent stem (iPS) cells have also become an attractive option for regeneration therapy because they possess self-renewal and pluripotent properties equivalent to those of ES cells [14]–[16]. Furthermore, the iPS cell technology enables the generation of individualized stem cells and thereby contributes to patient-oriented research, including developmental pathology, drug screening, and toxicity testing, which otherwise would be impossible in humans [17]–[18].

**Figure 1:**
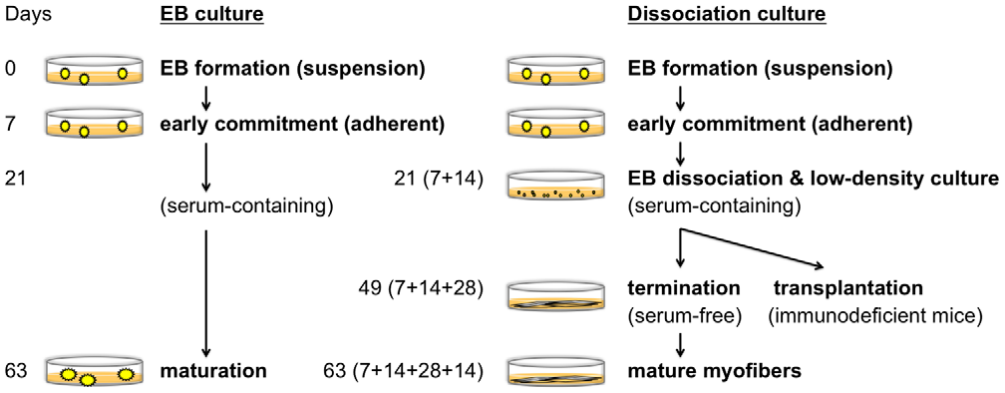
Schematic presentation of the differentiation protocol. Two different differentiation protocols were compared (Left: embryoid body (EB) culture, Right: Dissociation culture) and were exactly the same until the first 21 (7+14) days of culture. On the left side, EBs continued to be incubated in serum-containing medium without specific manipulation until the end of culture. On the right side, EBs and their outgrowth cells were dissociated and seeded onto collagen type I-coated tissue culture plates in serum-containing medium. The medium was changed to serum-free ITS medium on day 49 (7+14+28). In some experiments, the cells were harvested and used as donor cells for the transplantation assay at this time point.

Thus far, several groups have demonstrated myogenic differentiation from human ES (hES) cells with different induction strategies [10], [19]. The first approach is to induce lineage-specific differentiation by providing appropriate environmental factors, such as culture media, substrates, or cytokines. A classical approach is to induce myogenesis through the formation of three-dimensional cell aggregates called embryoid bodies (EBs), in which the differentiation processes of all 3 germ layers are recapitulated. Although it is an effective myogenic strategy employed for murine ES research [20], [21], this method had not been successfully executed in human ES cells because of various difficulties [22], until recently [23], [24]. However, the differentiation efficacy of these EB methods remains low and heterogeneous, and the potential of EB methods as a donor source for cell replacement therapy has not been investigated. Barberi et al. reported another two-dimensional culture protocol in which hES cells give rise to myogenic mesenchymal precursors. These precursor cells show efficient *in vitro* differentiation into skeletal myotubes and stable engraftment capacity *in vivo* [25], [26]. However, from a practical perspective, this approach is difficult to use because it requires repetitive cell sorting to obtain a population of homogenous myogenic mesenchymal progenitors. Another approach to achieve myogenic differentiation is the use of genetic modification to directly activate myogenic signaling pathways. Forced expression of *MyoD* is known to transform fibroblasts to skeletal muscle cells [27], and is also applicable to murine ES cells [28]. Darabi et al. demonstrated that the forced expression of *PAX3* successfully induced paraxial mesodermal and subsequent myogenic differentiation in murine ES cells [29], and the induction of *PAX7* efficiently induced myogenesis both in human ES and iPS cells [30]. These methods are indeed effective myogenic strategies. However, genetic modification is accompanied by various concerns, such as potent tumorigenesis.

**Figure 2:**
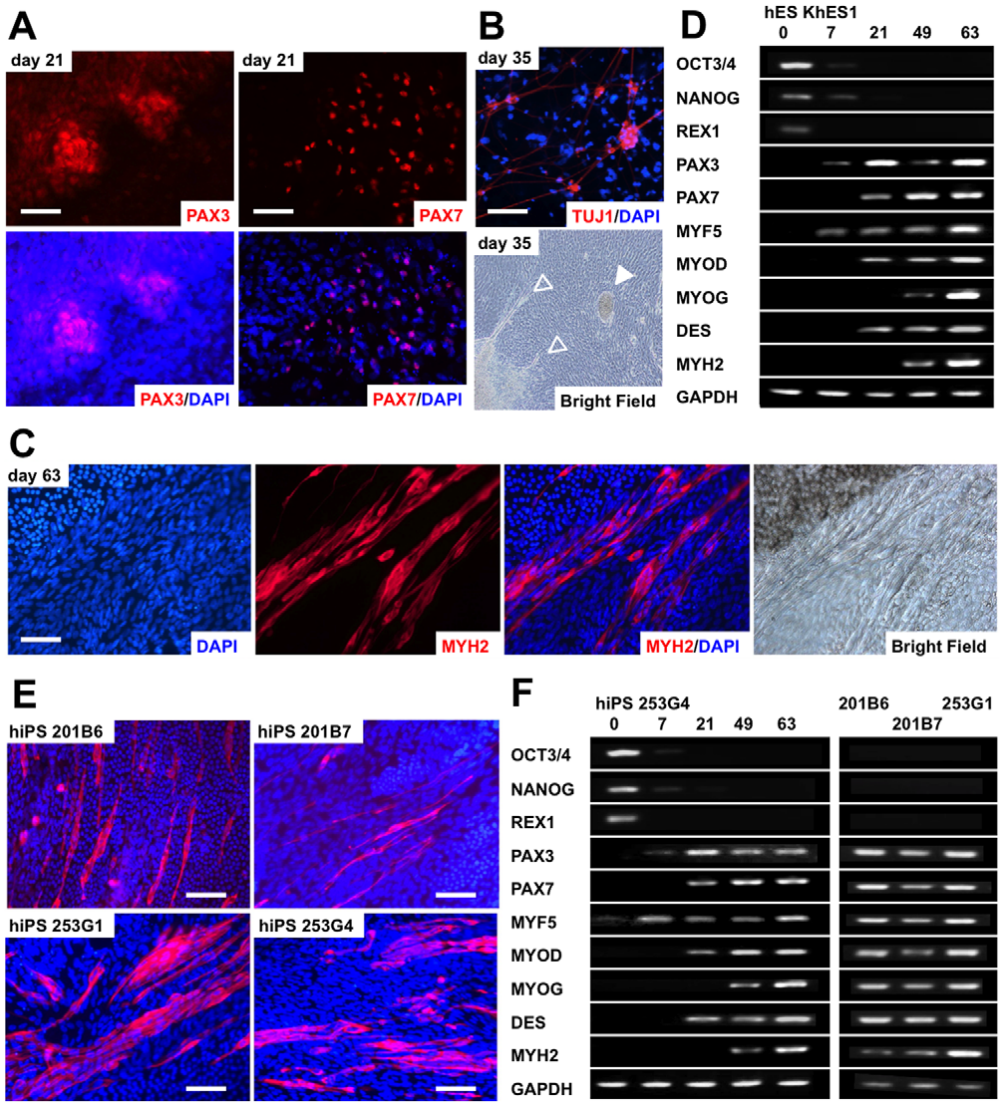
Skeletal muscle development from human embryonic stem (**hES**) **and human induced pluripotent stem** (**hiPS**) **cells by the EB culture method.** (A) PAX3- and PAX7-positive nuclei emerged in the proximal area of the embryoid body (EB)-outgrowth cells derived from hES KhES1 cells. (B) Simultaneous derivation of neural and cardiac cells in the EB-outgrowth cells derived from hES KhES1 cells. Upper: TUJ1-positive neural cells observed on day 7+28. Lower: Neural cells (outlined arrowheads) and colonies of beating cardiomyocytes (white arrowhead) appeared on day 7+28. (C) Skeletal myosin-positive myofibers in the EB-outgrowth cells derived from human embryonic stem (hES) KhES1 cells detected on day 7+42. (D) Sequential analysis of undifferentiated and skeletal myogenesis-related gene expression by semi-quantitative RT-PCR. (E) Skeletal myosin-positive fibers from human induced pluripotent stem (hiPS) cells. Four hiPS cell-lines were used. hiPS 201B6 on day 7+105, hiPS 201B7 on day 7+105, hiPS 253G1 on day 7+77, and hiPS 253G4 on day 7+56. (F) Sequential analysis of undifferentiated and skeletal myogenesis-related gene expression by semi-quantitative RT-PCR. In (A-C) and (E), antibodies were visualized using Cy3 (red). Nuclei were counterstained with DAPI (blue). Scale bars  = 100 µm.

We previously reported on an EB-based culture method that induces efficient myogenesis from both murine ES and iPS cells, and described the purification of satellite-like cells and their persistent engraftment capacity *in vivo* [31], [32]. The aims of this study were to 1) establish a novel *in vitro* culture system to induce efficient myogenesis from both human ES and iPS cells by modifying our EB-based method, without introducing any additional genes; and 2) investigate the capacity of the differentiated cells to engraft and repair damaged muscles *in vivo* to clarify whether they are an appropriate donor source in muscle regenerative medicine. Here, we describe a novel culture method for efficient myogenesis by using selective expansion of myogenic mesenchymal cells *in vitro* and demonstrate their capacity to engraft *in vivo*.

**Figure 3:**
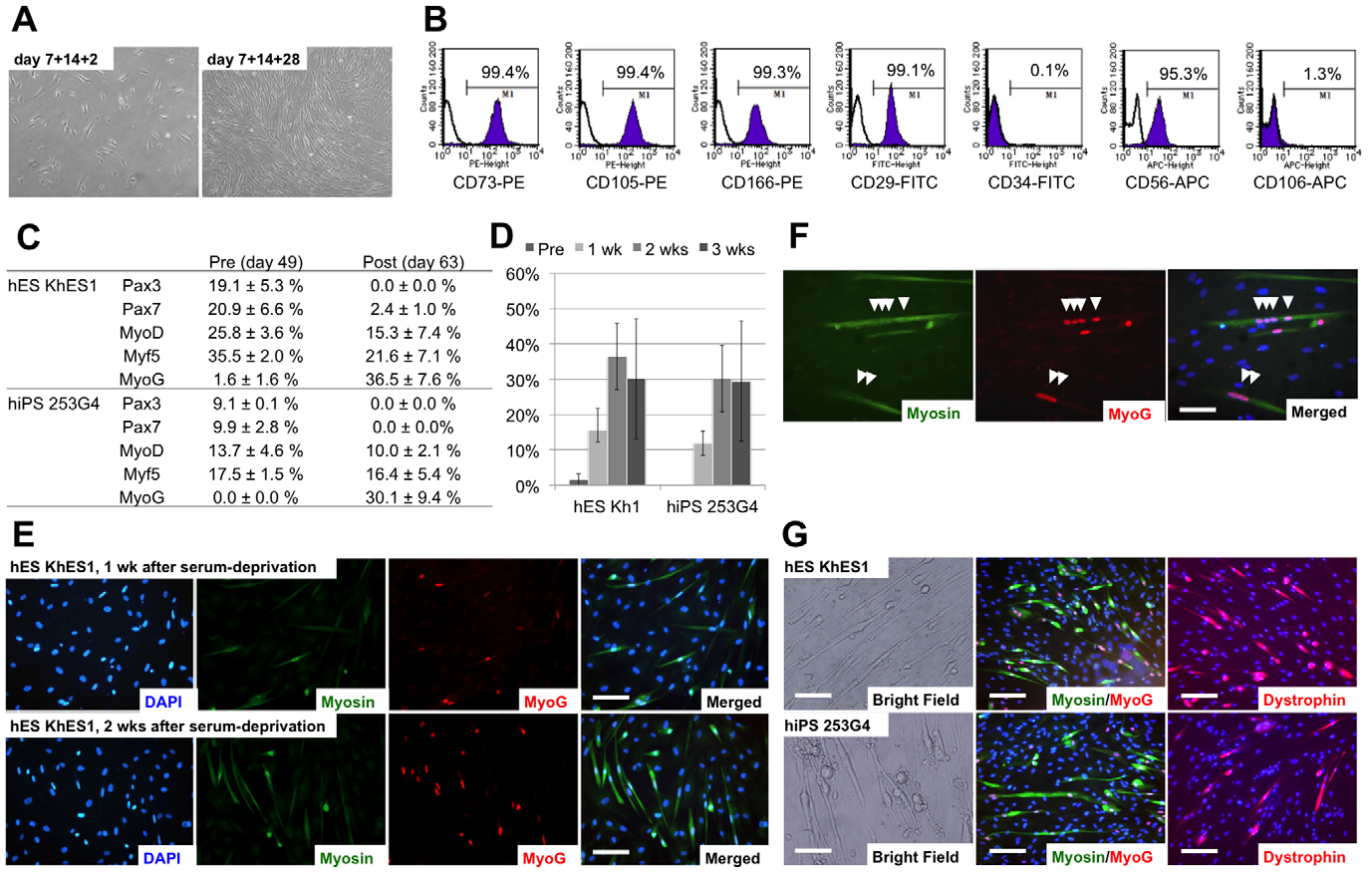
Characterization and differentiation of the derived myogenic mesenchymal cells. (A) Morphology of the derived myogenic mesenchymal progenitors 2 days (day 7+14+2) and 4 weeks (day 7+14+28) after replating. Homogeneous spindle-shaped fibroblastic cells were observed. (B) Surface marker analysis of myogenic mesenchymal progenitors. Representative data from KhES1 differentiation are shown. Note that CD56 in addition to mesenchymal markers CD73, CD105, CD166, and CD29 was exclusively expressed. (C) Changes in the expression of myogenic markers were analyzed by immunofluorescence. The number of Cy3-positive nuclei was divided by the total number of nuclei stained by DAPI. The expression of myogenic progenitor markers decreased after exposure to serum-free medium, whereas the number of MYOG-positive cells substantially increased after serum deprivation. (D) Changes in the number of MYOG-positive nuclei were observed up to 3 weeks after serum deprivation. hES/iPS-derived myofibers tended to detach from tissue culture plates during long-term culture in serum-free medium. (E) Serum deprivation increased the number of skeletal myosin-positive fibers and MYOG-positive nuclei for more than 2 weeks. KhES1 was used in this figure. (F) Multinucleated myofibers denoted by MYOG myogenin-positive nuclei aligned in skeletal myosin-positive fibers. (G) Morphology of mature myofibers, which were stained with skeletal myosin, MYOG, and dystrophin, from both KhES1 and 253G4 cells. Skeletal myosin was visualized with fluorescein isothiocyanate (FITC) (Green), myogenin was visualized with Cy3 (red), and nuclei were counterstained with DAPI (blue). Scale bars  =  (C, E) 100 µm, (D) 50 µm.

## Materials and Methods

### Maintenance of human ES and iPS cells

The hES cell line KhES1 was a kind gift from Dr. Norio Nakatsuji (Kyoto University, Kyoto, Japan) [33]. The hiPS cell lines 201B6, 201B7, 253G1, and 253G4 were established from human dermal fibroblasts by retrovirus-mediated transfection of 4 (201B6 and 201B7) or 3 (253G1 and 253G4) transcription factors (Oct3/4, Sox2, and Klf4, with or without c-Myc) [15], [16]. The human ES and iPS cell lines were maintained on mitomycin-C (Kyowa Hakko Kirin, Tokyo, Japan)-treated SNL feeder cells in human ES cell maintenance medium [hESM: DMEM/F12 (Sigma-Aldrich, St. Louis, MO, USA) supplemented with 20% Knockout^®^ Serum Replacement (Invitrogen, Carlsbad, CA, USA), 1% nonessential amino acid solution (Invitrogen), 5 mM sodium hydroxide solution, 100 µM 2-mercaptethanol, 2 mM L-glutamine] with 5 ng/ml basic fibroblast growth factor (R&D Systems, Minneapolis, MN, USA). The culture medium was changed daily. Colonies were passaged every 4 or 5 days.

**Figure 4:**
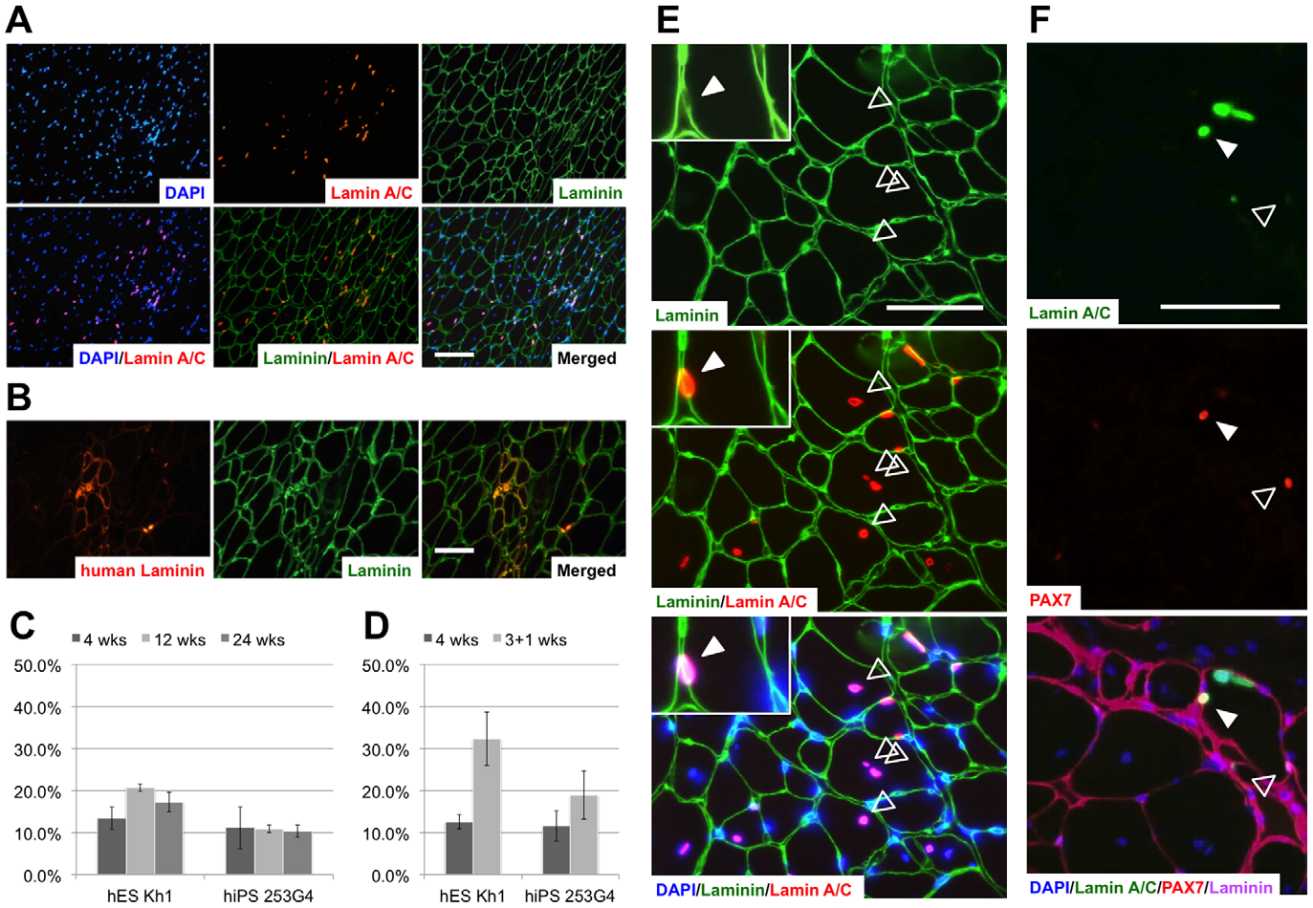
Engraftment of myogenic progenitors in damaged muscles of immunodeficient mice. (A) Human nuclei labeled with human-specific lamin A/C localized mainly inside muscle fibers surrounded by laminin. (B) Muscle reconstruction by transplanted human cells was demonstrated by the detection of human-specific laminin-alpha 2. (C) The proportion of myofibers containing human nuclei at 4, 12, and 24 weeks after transplantation. (D) The proportion of myofibers containing human nuclei in reinjured (3+1 weeks) and in non-reinjured mice (4 weeks) at 4 weeks after transplantation. In C and D, data are presented as the mean ± standard deviation. (E) Distribution of the transplanted cells at 24 weeks after transplantation. Typical central nuclei of human origin were observed (outlined arrowheads). Some human cells located within the lamina rara beneath the basal lamina, indicating engraftment of the transplanted cells into a satellite cell compartment (white arrowhead). (F) Triple-staining for human Lamin A/C, PAX7, and pan-Laminin clearly demonstrated the existence of PAX7-positive human nuclei indicating the transplanted cells engrafted as satellite cells (white arrowhead). Human lamin A/C-negative host satellite cells were also detected (outlined arrowhead). Laminin was stained by a polyclonal antibody that recognizes both human and murine laminin, and was subsequently visualized with fluorescein isothiocyanate (FITC) (Green); human lamin A/C and human-specific laminin, with Cy3 (red). Nuclei were counterstained with DAPI (blue). Scale bars  =  (A) 100 µm, (B) and (E) 50 µm.

**Table 1:** Transplantation efficacy demonstrated by the percentage of human nuclei in the transplanted muscles.

		KhES1	Average	SD		253G4	Average	SD	
*	4 weeks #1–1	15.1%				17.3%			
*	4 weeks #1–2	13.0%				18.6%			
*	4 weeks #2–1	8.1%				7.4%			
*	4 weeks #2–2	16.1%				6.1%			
**	4 weeks #3–1	12.6%				7.2%			
**	4 weeks #3–2	15.8%	13.5%	2.7%		10.3%	11.1%	5.0%	
*	12 weeks #1–1	26.5%				11.7%			
*	12 weeks #1–2	29.3%				10.3%			
*	12 weeks #2–1	13.5%				†			
*	12 weeks #2–2	16.5%				9.5%			
**	12 weeks #3–1	17.6%				11.7%			
**	12 weeks #3–2	ND	20.7%	6.1%		11.5%	10.9%	0.9%	
*	24 weeks #1–1	16.2%				12.2%			
*	24 weeks #1–2	13.9%				9.3%			
*	24 weeks #2–1	16.3%				9.6%			
*	24 weeks #2–2	20.9%				8.1%			
**	24 weeks #3–1	16.3%				11.4%			
**	24 weeks #3–2	19.6%	17.2%	2.3%		11.1%	10.3%	1.4%	
**	4 weeks #4–1	12.3%				6.9%			
**	4 weeks #4–2	14.6%				11.9%			
**	4 weeks #4–3	10.4%	12.5%	1.7%		15.7%	11.5%	3.6%	
**	3+1 weeks #4–1	30.4%				ND			
**	3+1 weeks #4–2	25.8%				13.0%			
**	3+1 weeks #4–3	40.7%	32.3%	6.3%	P<0.05	24.7%	18.9%	5.8%	NS

The number of cells of human origin was divided by the number of total nuclei stained by DAPI. The result also appears in Figures 4C and 4D. SD: standard deviation. ND: not detected. *, ** indicate the cell numbers transplanted at the site: * 5.0×10^5^ cells/site, ** 1.0×10^5^ cells/site. †: One mouse transplanted with 253G4-derived cells died accidentally before analysis. NS: statistically not significant.

The human ES and iPS cell lines were used in conformity with The Guidelines for Derivation and Utilization of Human Embryonic Stem Cells of the Ministry of Education, Culture, Sports, Science and Technology, Japan.

### Skeletal muscle differentiation

EB culture (Figure 1, Left): Undifferentiated human ES and iPS cells were trypsinized and floated in hESM as clusters for 7 days to form EBs. These EBs were then transferred to 0.1% gelatin-coated tissue culture plates and cultured in ITS medium [DMEM supplemented with ITS-X (Invitrogen), nonessential amino acids (Invitrogen), Glutamax^®^ supplement (Invitrogen), and 100 µM 2-mercaptoethanol (Wako, Osaka, Japan)] for 14 days. Afterwards, the medium was changed to skeletal muscle induction medium [SkIM: high-glucose DMEM supplemented with 10% fetal calf serum (FCS; Invitrogen), 5% horse serum (HS; Sigma), nonessential amino acids (Invitrogen), and 100 µM 2-mercaptoethanol (Wako)]. The cells were analyzed on days 7, 21, 35, 49, 63, 84, and 112. The medium was changed every 3 to 4 days.

Dissociation culture (Figure 1, Right): In some experiments, EBs and their outgrowth cells were treated with 0.25% trypsin/EDTA on day 21 and seeded onto collagen type I-coated tissue culture plates (BD Bioscience, Bedford, MA, USA) at a density of 3000 cells/cm^2^ in SkIM. On day 49, the medium was changed to ITS medium. For obtaining mature myofibers, cells were cultivated for up to 70 days. The differentiated cells were analyzed on days 7, 21, 49, 56, 63, and 70.

### Complementary DNA (cDNA) synthesis and reverse transcription-polymerase chain reaction (RT-PCR)

Cells were trypsinized on the indicated days of differentiation. Total RNA samples were extracted using silica gel membrane-based spin columns (RNeasy Mini-Kit^®^, Qiagen, Valencia, CA, USA). cDNA samples were synthesized using the Ominscript-RT Kit^®^ (Qiagen) and used for subsequent PCR. All procedures were performed according to the manufacturer’s instructions. The cDNA templates were initially denatured at 94°C for 5 min, followed by 35 amplification reactions consisting of 94°C for 30 seconds (denaturing), 55–60°C for 30 seconds (annealing), and 72°C for 60 seconds (extension), with a final extension at 72°C for 7 min. Oligonucleotide primers were designed for Oct3/4, Nanog, Pax3, Pax7, Myf5, MyoD, Myogenin, Desmin, TUBB3, TNNI3, and GAPDH. The primer sequences and temperature settings are described in supplemental materials (Table S1).

### Flow cytometric (FCM) analysis

Staining procedures, FCM analysis, and cell sorting were performed as described previously [31], [32]. Cells trypsinized on the indicated days of differentiation were stained with phycoerythrin- or allophycocyanin-conjugated primary antibodies (Abs). Abs used for FCM analysis included mouse anti-CD73, anti-CD105, anti-CD166, anti-CD34, anti-CD56, anti-CD106 (BD Bioscience), and anti-CD29 (Beckman Coulter, Brea, CA, USA). Dead cells were excluded by propidium iodide (Sigma). Samples were analyzed using FACSCalibur (BD Bioscience) and Cell Quest software (BD Bioscience).

### Immunostaining assays

Immunofluorescence analyses were performed as previously described [31], [32]. Briefly, samples were fixed for 5 min in 4% paraformaldehyde (PFA) and then permeabilized with 0.1% Triton X-100 in phosphate-buffered saline (PBS) for 10 min. After incubation in 2% skim milk for 1 h at room temperature to block nonspecific antibody binding, cells were incubated with primary antibodies for 1 h at room temperature. The primary antibodies used in this study were as follows: mouse anti-Pax3 (R&D Systems, Minneapolis, MN, USA), mouse anti-Pax7 (R&D Systems), rabbit anti-Myf5 (Santa Cruz Biotechnology, Inc., Santa Cruz, CA, USA), mouse anti-MyoD1, mouse anti-myogenin (Dako, Carpinteria, CA, USA), mouse anti-fast twitch myosin heavy chain (MYH2) (MY32; Zymed Laboratories, San Francisco, CA, USA), rabbit anti-skeletal myosin (Sigma), mouse anti-dystrophin (MANDRA1; Sigma), rabbit anti-laminin (Dako), mouse anti-human merosin (Laminin alpha 2), mouse anti-human lamin A/C (Novocastra Laboratories, Newcastle-upon-Tyne, UK), mouse anti-beta-tubulin III (TUJ1) (Sigma), and rabbit anti-glial fibrillary acidic protein antibody (GFAP) (Sigma). After washing twice with PBS, samples were incubated with secondary antibodies for 1 h at room temperature. The secondary antibodies used in this study were Cy3-conjugated anti-mouse IgG (Jackson ImmunoResearch Laboratories Inc., West Grove, PA, USA) and fluorescein isothiocyanate (FITC)-conjugated anti-rabbit IgG (Jackson ImmunoResearch). DAPI was used for nuclear staining. These samples were then examined using fluorescent microscopes (FluoView System; Olympus, Tokyo, Japan). Photographs were acquired with an Axio-Cam (Carl Zeiss Vision, Hallbergmoos, Germany). To quantify myogenic transcription factor-positive cells, we counted the number of Cy3-positive nuclei and divided this value by the total number of nuclei stained by DAPI.

For triple staining, we modified the protocol described by Darabi and his colleagues [30]. Briefly, samples were fixed for 20 min in 4% PFA, followed by permeabilization using 0.3% Triton X-100 in PBS. After incubation in blocking solution consisting of a mixture (1∶1) of 3% bovine serum albumin (Invitrogen) and MOM blocking agent (Vector Laboratories Inc., Burlingame, CA, USA) for 1 h at room temperature, the slides were incubated with mouse anti-Pax7 (R&D Systems). After washing twice with PBS, samples were incubated with alexa-fluor 555 conjugated anti-mouse IgG (Invitrogen) for 30 min. After washing twice, the slides were blocked using the same solution for 1 h at room temperature. Subsequently, the slides were incubated with mouse anti-human Lamin A/C (Novocastra) and rabbit anti-laminin (DAKO) for 1 h at room temperature, followed by incubation with alexa fluor 488-conjugated anti-mouse IgG and alexa fluor 647-conjugated anti-rabbit IgG (Jackson Immunoresearch). The nuclei were counterstained using DAPI and analyzed by AS-MDW system (Leica Microsystems GmbH, Wetzlar, Germany).

### Transplantation

Eight-week-old male immunodeficient NOD/Shi-scid/IL-2Rγ^null^ (NOG) mice (Central Laboratories for Experimental Animals, Kanagawa, Japan) [34] were used as host mice to avoid immunological rejection. Host tibialis anterior (TA) muscles were bilaterally injected with 50 µL of 10 µM cardiotoxin (CTX; Latoxan, Valence, France) to induce muscle degeneration 24 h before transplantation. These mice were systemically irradiated to block endogenous muscle regeneration. Irradiation was delivered in 2 fractions of 1.2 Gy, a sub-lethal dose for NOG mice. The cells (1.0×10^5^ or 5.0×10^5^) were suspended in 20 µL of medium and directly injected into the pre-damaged left TA muscles. The same amount of medium was injected into the right TA muscles, which served as the control. Mice were sacrificed at 4, 12, and 24 weeks after transplantation and subjected to immunohistological analyses. In some experiments, the transplanted mice were re-injured by CTX injection at 3 weeks after transplantation and analyzed 1 week later to determine the regenerative capacity of the engrafted cells. For the immunohistological assay, the isolated muscles were frozen in isopentane cooled in liquid nitrogen. The frozen specimens were sectioned with a cryostat (CM1850; Leica Microsystems, Wetzlar, Germany) and analyzed as described above. All animal handling procedures were followed according to the Guide for the Care and Use of Laboratory Animals published by the U.S. National Institutes of Health (NIH Publication No. 85–23, revised 1996) and the Guidelines of the Animal Research Committee of the Graduate School of Medicine, Kyoto University. This work was approved by the Animal Research Committee of the Graduate School of Medicine, Kyoto University. Mice were sacrificed by cervical dislocation. All painful procedures including cervical dislocation were performed under anesthesia. In immunofluorescence analyses, the percentage of human nuclei in total nuclei was counted.

### Statistics

Statistical analyses were performed using the unpaired Student’s *t* test, and a value of *P*<0.05 was considered to be statistically significant.

## Results

### Myogenic induction by human ES and iPS cells in EB outgrowth culture

To develop an efficient differentiation protocol for inducing skeletal myogenesis from human ES and iPS cells, we adapted our previously established EB-based method that induces myogenic differentiation from murine ES and iPS cells [31], [32]. EBs were formed by suspension in hESM for 7 days and then plated onto 0.1% gelatin-coated tissue culture plates. Attached EBs were cultured in serum-free medium for an additional 14 days and then in differentiation medium containing 10% FCS and 5% HS until day 112 (7+14+91) of differentiation. When EBs attached to the plates, the cells migrating out from the EBs formed a single layer, which we termed an EB-outgrowth (EB-OG). Immunostaining for PAX3 and PAX7, which mark early myogenic progenitors, showed that clusters of PAX3- and PAX7-positive cells were randomly distributed at day 21 (Figure 2A). Skeletal myosin (MYH)-positive multinucleated myofibers had appeared within most of the attached EBs at day 63 (Figure 2C) and were randomly distributed in the crowded EB-OG. Some attached EBs simultaneously contained TUJ1-positive neural cells (Figure 2B) and colonies of beating cardiomyocytes (Figure 2B), as previously observed in studies on murine EB differentiation [20], [21].

We then analyzed the expression of genes associated with skeletal myogenesis, including *PAX3, PAX7,* desmin (*DES*), and the muscle regulatory factors *MYF5*, *MYOD*, and *MYOG* (Figure 2D). The expression of *PAX3* was first detected on day 7, increased at day 21 (7+14), and decreased thereafter. *PAX7*, which is essential for the specification of muscle progenitors [3]-[5], [35], [36], was detected at day 21 (7+14), but its expression was highly variable. *MYOD* and *DES* were first detected at day 21 (7+14), and their expression increased thereafter. *MYF5* was detected at day 7, and its expression was sustained throughout differentiation. The expression of *MYOG* was not detected until day 49 (7+42) but increased thereafter. In contrast, the pluripotent stem marker genes *OCT3/4*, *NANOG*, and *REX1* were down regulated during differentiation. Thus, the hierarchical expression of myogenic genes in this system was similar to the patterns observed during murine embryogenesis [37] and to those of the EB differentiation of murine ES and iPS cells [20], [31], [32].

To verify whether this protocol was applicable to hiPS cells, we used 4 iPS cell lines expressing either 4 (*OCT3/4*, *SOX2*, *NANOG*, and *MYC*) [15] or 3 (lacking *MYC*) retrovirally introduced transcription factors [16]. The EBs from all examined hiPS cell lines contained MYH-positive myofibers, confirming the myogenic potential of hiPS cells (Figure 2E). The expression pattern of myogenic markers was quite similar to that of ES cells (Figure 2F), although the differentiation efficacy was different among the cell lines.

Thus, EB-OG successfully induced myogenesis from both human ES and iPS cells, thereby simulating the temporal patterning of embryogenesis. However, the EB-OGs were so heterogeneous that we could not distinguish the differentiated muscle lineage cells from other lineage cells. Most of the skeletal muscle lineage cells appeared as clusters in crowded EB-OGs and never appeared as a homogeneous population.

### Selective expansion of myogenic mesenchymal cells from dissociated EB outgrowth culture

Because mature myofibers could not clearly be distinguished in the overcrowded EB-OG cells, we decided to purify the myogenic cells from the other EB-OG cells. We hypothesized that the separation of the myogenic cells might allow efficient myogenesis if these other lineage cells were disturbing skeletal myogenesis in the EBs. Hence, the differentiated EB-OG cells were dissociated into single cells and re-cultured on collagen type I-coated plates on day 21 (7+14) when the clusters of PAX3- and PAX7-positive myogenic progenitors were detected. When the dissociated cells were replaced onto collagen type I-coated plates, cells with a uniform spindle, fibroblastic morphology developed selectively during the next 4 weeks (Figure 3A). FCM analysis showed that the generated cells exclusively expressed the mesenchymal markers CD73, CD105, CD166, and CD29, whereas CD34 and CD106 were hardly detected. CD56 (NCAM; neural cell adhesion molecule) was also highly expressed (Figure 3B, Figure S1). A low-density culture of dissociated cells on collagen type I was crucial for selective development of myogenic cells; neural and cardiac cells were not detected in this dissociation protocol (Figure S2).

Quantification of the expression of the myogenic progenitor markers MYOD, MYF5, and PAX7 increased during cell proliferation and peaked around day 49 (7+14+28) in this dissociation protocol. Since serum deprivation is known to enhance further maturation of myogenic cells, we switched from serum-containing SkIM to serum-free ITS medium to induce terminal muscular differentiation. Following serum deprivation, the proportion of myogenic progenitor marker-positive nuclei decreased immediately, whereas that of MYOG-positive nuclei dramatically increased (Figure 3C–3E). The increased expression of skeletal myosin-positive fibers and the increased prevalence of MYOG-positive nuclei were maintained up to 3 weeks (Figure 3C, 3D).

Both hES (KhES1) and hiPS (253G4) cell-derived cells fused together and produced multinucleated myofibers that were positive for skeletal myosin and dystrophin (Figure 3F, 3G). These myofibers occasionally showed spontaneous contraction, indicating the maturation of skeletal muscle cells. Hence, the results showed that this multi-step culture method selectively isolated myogenic mesenchymal population from dissociated EB culture, and that these myogenic mesenchymal cell populations could terminally differentiate into functionally mature myofibers *in vitro*.

### Transplantation in immunodeficient NOG mice

Next, we examined the *in vivo* myogenic potential of human ES and iPS cell-derived myogenic mesenchymal cells by transplanting them into the injured muscles of NOG mice. As myogenic progenitor markers were strongly expressed at day 49 (7+14+28), the cells were trypsinized again and used as donor cells. To avoid immunological rejection, we used immunodeficient NOG mice as the hosts.

Four weeks after transplant, human-specific lamin A/C-positive nuclei were detected in TA muscles (Figure 4A). Human lamin A/C-positive and lamin A/C-negative nuclei were co-localized in the same muscle fascicle and had formed hybrid myotubes (Table 1, Figure 4A). Furthermore, the detection of human-specific merosin (laminin alpha 2) proved that the transplanted cells had not only survived within muscle fibers but had also produced human protein around the muscle fibers into which they had integrated (Figure 4B). Thus, the myogenic mesenchymal cells derived here engrafted and functioned to repair damaged muscles.

We also investigated the log-term outcome of the myogenic mesenchymal cell transplant. Human lamin A/C-positive nuclei were continuously observed in the damaged muscles until 24 weeks after transplantation (Table 1, Figure 4C). Moreover, the presence of typical central nuclei of human origin strongly suggested that transplanted cell-related muscular regeneration occurred even at 24 weeks after transplantation (Figure 4E). Although most human cells were detected within muscle fascicles, some of them resided within the lamina rara, which is consistent with the location of satellite cells (Figure 4E). These human nuclei were also positive for PAX7, confirming the contribution of the transplanted cells to the satellite cell fraction (Figure 4F). Under macroscopic and histological examination, teratoma formation was not observed up to 24 weeks.

Finally, to assess whether the engrafted myogenic mesenchymal cells could proliferate in response to subsequent damage, we employed a second injury model, as previously reported [31], [32]. Three weeks after transplantation, CTX was directly injected into the transplanted muscles. The proportion of human nuclei was higher in re-injured mice than that in non-re-injured mice at 4 weeks after transplantation in the experiment using hES cells, whereas no statistical significance was observed in the hiPS cells. (Table 1, Figure 4D).

Collectively, our results indicated that human ES and iPS cell-derived myogenic mesenchymal cells engrafted effectively, integrated into the satellite cell fraction, and contributed to the long-term reconstruction of the damaged muscle tissue without exhaustion.

## Discussion

Recent remarkable advances in human ES and iPS cell technology have increased the demand for the clinical application of these cells and their use in basic research. The excellent work by Barberi et al. clearly demonstrated the myogenic capacity of hES cells both *in vitro* and *in vivo* [26]; however, their strategy to obtain myogenic populations included repetitive cell sorting procedures, which would hamper practical use. The other approach to induce myogenesis from hES cells is to form EBs; however, the EB-based methods inevitably contain cells from other lineages. Ideally, homogeneous populations of the desired cell types should be generated using a simple method that does not involve complicated procedures.

In this report, we modified our murine ES and iPS cell culture system to develop a new EB-based culture method that can successfully induce mature skeletal muscle cells from hES cells. An advantage of this system is that selective expansion of myogenic mesenchymal cells can be accomplished by simple manipulation.

Moreover, this culture system is applicable to hiPS cells. Darabi and his colleagues recently reported successful skeletal muscle induction from hiPS cells by *PAX7* overexpression [30]. However, skeletal myogenesis from hiPS cells without genetic manipulation has not been previously reported. Our results facilitate the further use of hiPS cells in muscle regenerative medicine, which extends the possibilities of hES technology to clinical applications, such as patient-oriented research and autologous transplantation [10], [17]–[19].

We did not employ cell sorting in this method. Several reports have demonstrated that multipotent mesenchymal cells can be generated by long term EB culturing or repetitive passaging [38], [39]. Meanwhile, some subpopulations of mesenchymal stem cells (MSCs) are considered to possess myogenic potential. We re-plated dissociated EBs on several coating materials (Figure S2), and derived the immunophenotypically homogeneous mesenchymal cell population. Surface marker analyses demonstrated that the generated cells were uniformly positive for mesenchymal stem cell (MSC)-related surface antigens, including CD73, CD105, CD166, and CD29. However, the generated cells were different from MSCs because of the high expression of CD56. CD56 is known to be expressed in embryonic skeletal muscles and satellite cells [9], [11], and was used for the prospective isolation of myogenic progenitors from hES cell-derived MSCs in a previous report [26]. These reports also supported the existence of the myogenic mesenchymal cells generated here. Further study will be necessary to characterize the derived cells in terms of their multipotency, including their osteogenic, chondrogenic, and adipogenic activities.

The homogenous mesenchymal population with myogenic potential developed in our culture system; these can be used for both *in vitro* and *in vivo* studies. By depleting serum, the transition from myogenic progenitors to mature myotubes can easily be observed during culture. In combination with the recent advancements in creating disease-specific hiPS cell lines, this culture system will be a powerful experimental tool for disease studies and the screening of drugs for various types of muscle diseases, both of which have been difficult to conduct because of the significant challenge in obtaining sufficient materials.

Furthermore, we demonstrated the *in vivo* myogenic capacity of derived cells. When transplanted in NOG mice, the engrafted cells contributed to satellite cell fraction and reconstructed the injured muscles up to at least 6 months without rejection. Moreover, the integrated cells showed a more robust proliferation in response to subsequent injury. Myogenic progenitors with such re-proliferation capacity *in vivo* have not been reported in previous studies [22]–[26], [29], [30], and can be a feasible cell source for muscle regenerative medicine, since long-lasting muscle repair will be required for patients with genetic muscle diseases. The risk of teratoma formation was eliminated by selective culture conditions, which is also suitable for clinical settings.

The underlying mechanisms of the selective proliferation of myogenic cells in our culture system remain unresolved. The initial EB-OG culture simultaneously contained neuronal and cardiac cells in addition to muscle lineage cells. Given that re-plating the dissociated cells at low cell density was a crucial step for effective myogenic differentiation, direct cell-to-cell contact or soluble factors from other cells or cell lineages might have negatively affected the selective development of muscle lineage cells. Otherwise, simple cell selection through the capacity to adhere to collagen type I-coated plates or differences in proliferation potential enriched the myogenic mesenchymal population in the residual cells (Figure S2).

In conclusion, we have reported a novel culture system that induces homogenous myogenic mesenchymal cells from both human ES and iPS cells. This method is a modification of the widely used EB method and is accomplished using a simple procedure. To date, there still are few reports describing human ES and iPS cell myogenic differentiation. This system encourages further utilization of human ES and iPS cells in skeletal muscle research, which could not otherwise be conducted in humans, and will provide a promising therapeutic approach for various muscle diseases.

## Supporting Information

Figure S1 **Surface marker changes with or without EB dissociation.** Surface marker of the differentiating EBs without dissociation (left) and the cells after EB dissociation and re-plating (right). The mesenchymal stem cell marker, CD73, increased during continuous EB culture, but much homogeneous population was obtained at days 7+14+28 by EB dissociation. Representative histograms are shown. The experiments were conducted 3 times, and mean scores and standard deviations were calculated. SD: standard deviation.
(TIF)

Figure S2 **Elimination of other lineage cells by low-cell-density culture on collagen type I.** The results demonstrated that low-density culture on collagen type I eliminated neural and cardiac cells. (A) RT-PCR for TUBB3, TNNI3, and Desmin expression at different cell densities. RT-PCR result indicated contamination of neural and cardiac cells at high-density culture at variable levels. Laminin and poly-D-lysin preferentially captured many more neurogenic cells, whereas others did not. At low-density culture, 0.1% gelatin, laminin, and poly-D-lysin could not support cell proliferation. A heterogeneous non-mesenchymal population was obtained on collagen type IV. When cultured on Matrigel^®^, the results obtained were similar to that obtained using collagen type I. (B) TUJ1- or GFAP-positive cells observed at high-cell-density culture on collagen type I. At high cell density, the cells reached confluence at several days after re-plating. Upper: 7 days after re-plating. Lower: 28 days after re-plating. No neural cells were detected if cultured at a low-density.
(TIF)

Method S1 **Selection of myogenic cells by low-cell-density culture on collagen type I EBs were dissociated at day 21** (**7+14**) **of differentiation and re-plated onto different coating materials.** We used 0.1% gelatin, laminin, poly-D-lysin, collagen type I, collagen type IV, and Matrigel® (All from BD Bioscience) for the experiments. The cells from dissociated EBs were seeded at low- or high-cell-density (3,000 and 30,000 cells/cm^2^, respectively) and cultured for up to 28 days. Cells were analyzed at day 7 and 28 after re-plating by RT-PCR or immunostaining.
(DOCX)

Table S1 **The primer settings used for RT-PCR.**
(TIF)
