# Supplementary figures and images for: Selective Development of Myogenic Mesenchymal Cells from Human Embryonic and Induced Pluripotent Stem Cells
Source: PLoS One. 2012 Dec 7;7(12):e51638. doi: 10.1371/journal.pone.0051638 (PMC3517512; doi:10.1371/journal.pone.0051638)

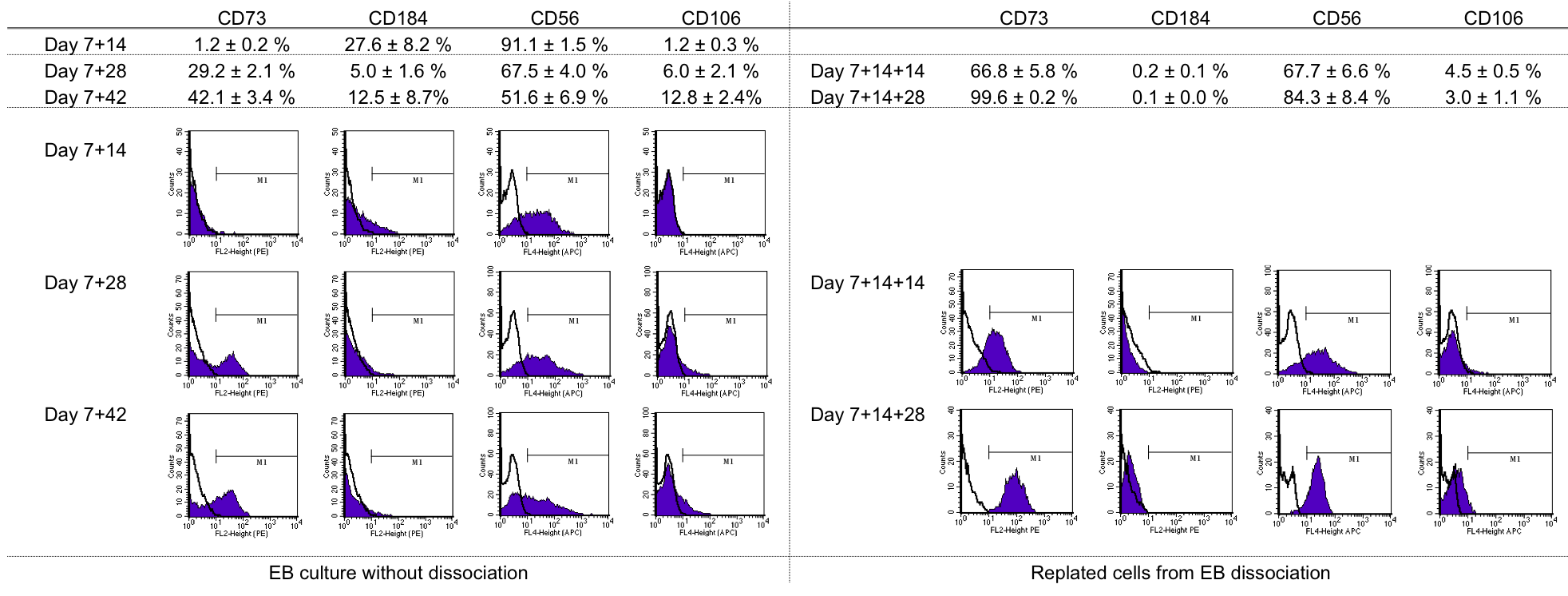

Supplement: Figure S1 — Surface marker changes with or without EB dissociation. Surface marker of the differentiating EBs without dissociation (left) and the cells after EB dissociation and re-plating (right). The mesenchymal stem cell marker, CD73, increased during continuous EB culture, but much homogeneous population was obtained at days 7+14+28 by EB dissociation. Representative histograms are shown. The experiments were conducted 3 times, and mean scores and standard deviations were calculated. SD: standard deviation. (TIF) [file pone.0051638.s001.tif]

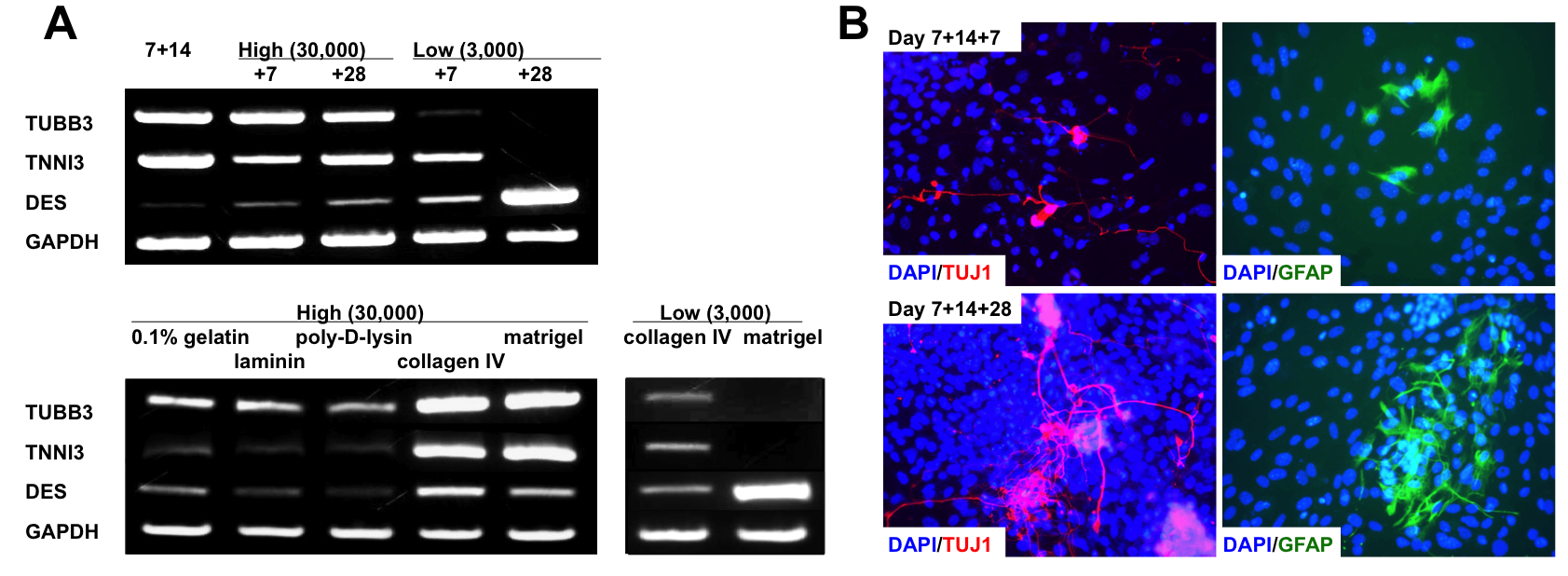

Supplement: Figure S2 — Elimination of other lineage cells by low-cell-density culture on collagen type I. The results demonstrated that low-density culture on collagen type I eliminated neural and cardiac cells. (A) RT-PCR for TUBB3, TNNI3, and Desmin expression at different cell densities. RT-PCR result indicated contamination of neural and cardiac cells at high-density culture at variable levels. Laminin and poly-D-lysin preferentially captured many more neurogenic cells, whereas others did not. At low-density culture, 0.1% gelatin, laminin, and poly-D-lysin could not support cell proliferation. A heterogeneous non-mesenchymal population was obtained on collagen type IV. When cultured on Matrigel®, the results obtained were similar to that obtained using collagen type I. (B) TUJ1- or GFAP-positive cells observed at high-cell-density culture on collagen type I. At high cell density, the cells reached confluence at several days after re-plating. Upper: 7 days after re-plating. Lower: 28 days after re-plating. No neural cells were detected if cultured at a low-density. (TIF) [file pone.0051638.s002.tif]

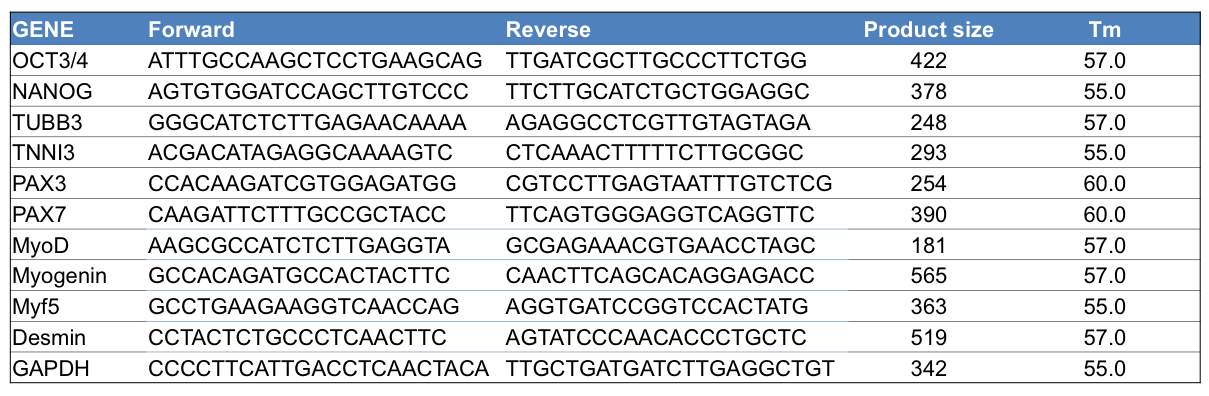

Supplement: Table S1 — The primer settings used for RT-PCR. (TIF) [file pone.0051638.s004.tif]
