# Supplementary material for: Selective Development of Myogenic Mesenchymal Cells from Human Embryonic and Induced Pluripotent Stem Cells
Source: PLoS One. 2012 Dec 7;7(12):e51638. doi: 10.1371/journal.pone.0051638 (PMC3517512; doi:10.1371/journal.pone.0051638)
Supplement: Method S1 — Selection of myogenic cells by low-cell-density culture on collagen type I EBs were dissociated at day 21 (7+14) of differentiation and re-plated onto different coating materials. We used 0.1% gelatin, laminin, poly-D-lysin, collagen type I, collagen type IV, and Matrigel® (All from BD Bioscience) for the experiments. The cells from dissociated EBs were seeded at low- or high-cell-density (3,000 and 30,000 cells/cm2, respectively) and cultured for up to 28 days. Cells were analyzed at day 7 and 28 after re-plating by RT-PCR or immunostaining. (DOCX) [file pone.0051638.s003.docx]

**Method S1**

Supplemental Method S1. Selection of myogenic cells by low-cell-density culture on collagen type I

EBs were dissociated at day 21 (7+14) of differentiation and re-plated onto different coating materials. We used 0.1% gelatin, laminin, poly-d-lysin, collagen type I, collagen type IV, and Matrigel® (All from BD Bioscience) for the experiments. The cells from dissociated EBs were seeded at low- or high-cell-density (3,000 and 30,000 cells/cm^2^, respectively) and cultured for up to 28 days. Cells were analyzed at day 7 and 28 after re-plating by RT-PCR or immunostaining.
